# Characterization of ectomycorrhizal fungal communities associated with tree species on an iron tailings deposit undergoing restoration

**DOI:** 10.1007/s11356-022-21690-0

**Published:** 2022-07-02

**Authors:** Wenxu Zhu, Changjun Ding, Keye Zhu, Weixi Zhang, Dejun Liang, XiaoJiang Wang, Aiping Li, Xiaohua Su

**Affiliations:** 1 College of Foresty, The University of Shenyang Agriculture, Dongling Road, Shenyang, China; 2 Present Address: State Key Laboratory of Tree Genetics and Breeding, Research Institute of Forestry, Chinese Academy of Forestry, Beijing, China; 3 Key Laboratory of Tree Breeding and Cultivation of State Forestry Administration, Research Institute of Forestry, Chinese Academy of Forestry, Beijing, China; 4 Liaoning Provincial Poplar Institute, Gaizhou, Liaoning China; 5 Inner Mongolia Academy of Forestry Sciences, Hohhot, Inner Mongolia China

**Keywords:** Mining, Reclamation, Ectomycorrhizal fungi, *Robinia pseudoacacia* L., Soil characteristics

## Abstract

Vegetation restoration is an effective method to improve the ecological environment of mine tailings, which has a profound impact on the potential ecological functions of soil fungal communities; yet, little is known about its beneficial effect on soil ectomycorrhizal fungal community. In this study, the responses of soil characteristics and soil ectomycorrhizal fungal community diversity and structure to different revegetation, as well as the contribution of soil factors to soil ectomycorrhizal community were investigated in Liaoning Province, China. As we anticipated, the presence of vegetation significantly improved most soil properties we studied. What’s more, compared to Korean pine (*Pinus koraiensis* Sieb. et Zucc.), Chinese poplar (*Populus simonii* Carr), and black locust (*Robinia pseudoacacia* L) could better improve soil total carbon, total nitrogen, total phosphorus, and available phosphorus. In addition, soil ectomycorrhizal fungal community diversity in black locust was greater than Korean pine and *Populus simonii*. Nonmetric multidimensional scaling analyses indicated that soil ectomycorrhizal community significantly differed depending on different revegetation types. Thus, these results indicated that black locust could be a suitable species for the revegetation of iron mine tailings. The study provided theoretical basis for ecological restoration of iron mine tailings using local plant species.

**Supplementary Information:**

The online version contains supplementary material available at 10.1007/s11356-022-21690-0.

## Introduction

China is one of the world’s most biodiversity countries and also a large exporter of commodities such as iron ore. Iron-mining activities have important economic and social benefits, despite having a significant impact on the landscape, soil basic characteristics, and soil microbial community (Skirycz et al. 2014). Iron-mining activities not only dramatically occupy and destroy a large amount of land, produce large amount of mine tailings, reduce soil organic matter stock, threat to the biodiversity and result in compromised ecosystem functions, but also pollute air environment and affect human health for a long time (Ngugi et al. 2018). From a long-term perspective of sustainable use of land resources and ecological environment protection, there is an urgent need for a feasible way to restore the degraded ecosystems (Wang et al. 2017).

In areas degraded by iron mining, natural regeneration is slow and often impossible due to the physical and chemical characteristics of the substrate (González-Alcaraz and van Gestel 2017), such as poor physical structure (Silva et al. 2007), alkaline pH (Wu et al. 2020), low water retention capacity, nutrient (N, P) deficiency (Wu et al. 2018a, b), and high metal stress (Lopez-Orenes et al. 2017; Wang et al. 2017). In addition, in degraded iron ore areas with the characteristic of low nutrient concentration and high density of the substrate, artificial vegetation restoration is a huge challenge (Rios et al. 2021; Wang et al. 2017). In recent years, the recovery of iron ore–mined areas can be achieved through a variety of hard engineering techniques, and revegetation has been considered to be a more efficient, economical, and environmentally sustainable remediation strategy compared to physical and chemical methods (Wang et al. 2017; Skirycz et al. 2014), as it could preserve the soil resource, improve soil structure, physico- and biochemical properties, biodiversity patterns, ecosystem functioning (Gastauer et al. 2018), soil microbial diversity (Ngugi et al. 2020; Xue et al. 2015), ultimately creating self-sustaining vegetation communities. Given that, the selected species for revegetation purposes must be able to thrive under these multi-stress scenarios (Peng et al. 2019), and the selection of suitable plants species is the first step for restoration of mine tailings.

In previous studies, many herbaceous (Mahdavian et al. 2017; Heckenroth et al. 2016) and woody plants (Luo et al. 2019; Siebielec et al. 2018), such as *Paspalum densum*, *Setaria parviflora* (Rios et al. 2017; Araújo et al. 2015), and *Robinia Pseudoacacia* (Deng et al. 2020a, b), have proven to be potential candidates for revegetating iron ore–mined areas. In addition, the integration of soil biological indicators with chemical and physical indicators is an important factor in the evaluation of soil quality and the recovery process (Silva et al. 2018). A number of mine-tailing reclamation findings have emphasized a strong association between the establishment of plant community and the abundance and composition of soil microbiota (Deng et al. 2020a; Mendez et al. 2008), mainly focusing on the bacteria (Deng et al. 2020a), fungi (Deng et al. 2020b), and arbuscular mycorrhizal fungi (AMF)(Wu et al. 2020; Prado et al. 2019). However, the links between plant taxa succession and their associated ectomycorrhizal fungal communities remain to be addressed.

Ectomycorrhizal (ECM) fungi have many beneficial ecological effects on host plants. They cannot only improve plant roots ability to absorb soil moisture and nutrients (Van and Hartmann 2016; Augé et al. 2007), enhance plant photosynthesis (Gong et al. 2013), but also directly synthesize or induce host plants to produce a variety of hormones (Fitze et al. 2005), thereby improving plant adaptation to various environmental stresses, driving critical ecosystem functions and then promoting the restoration and reconstruction of degraded ecosystem (Silva et al. 2018; Leal et al. 2016). Considering the importance of ECM fungi in soil ecosystem, it is important to understand the composition and diversity of ECM fungal communities at tailing sites, for the sake of mined land rehabilitation.

Iron ore in China is widespread and relatively concentrated. At present, there are five major areas of concentrated distribution of iron ore reserves in China, among which Anshan-Benxi iron mine wasteland in the northeast covers the largest area. Iron ore mining has made a great contribution to the regional economic development (Wilson 2012); however, serious environmental problems are caused by iron mining in China. Furthermore, with the implementation of national policies related to ecological civilization construction, it is imperative to carry out reasonable mine ecological restoration in Anshan-Benxi iron-mined area to ensure the harmonious development of society, economy, and environment. Therefore, the construction of green mines and ecological restoration should be actively promoted in the process of mining development (Sheoran et al. 2010). At present, considerable researches mainly focus on the different technologies for land reclamation and ecological restoration (Zhang 2018; Zhang et al. 2018), as well as the impact of ecological restoration on soil macro-animal communities (Liu et al. 2009) and soil microorganisms (Deng et al. 2020a) in iron-mining areas of Liaoning Province. However, information as to the ECM fungal communities associated with different revegetation is insufficient and therefore needed. Therefore, the objective of this study was to investigate whether the three native woody plants, including Korean pine, Chinese poplar, and black locust can improve the soil basic characteristics and soil ECM fungal community after 15 years of aided phytostabilization under field conditions. It is hypothesized that (1) vegetation restoration could promote the accumulation of soil nutrients; (2) ECM fungal community diversity and composition would exist significant difference among different vegetation restoration; (3) along with the restoration of vegetation, the remarkable abiotic changes were the accumulation of soil nutrients, which affect the shifts of ECM fungal communities. The findings of this study will be beneficial for the selection of suitable vegetation types to accelerate the vegetation restoration process in iron mine tailing.

## Materials and methods

### Site information

The study area is located in Dengta City, Liaoyang City, Liaoning province, China (40.74 N, 122.86 E), which is classified as north temperate continental climate with the feature of warm spring, hot summer, cool autumn, cold winter, four distinct seasons, rain in hot season, sufficient sunshine. The annual average temperature is 8.8℃, and the annual average frost-free period is 171 days. The rainfall is abundant, mainly in summer, with an average annual total rainfall of about 686.0 mm. Chinese pine (*Pinus tabuliformis*), *Larix gmelinii* (*Larix gmelinii* (Ruprecht) Kuzeneva), Korean pine, Chinese poplar, and black locust and elm (*Ulmus pumila*) are the main vegetation.

### Sample collection

The details of study area and plot setting were described in the study from Deng et al. (2020a). Prior vegetation before the mining was shrubbery. The selected restoration areas were first mined in 2001, and mining ended in 2006. With the proposal of the concept of lucid waters and lush mountains, mine abandoned land reclamation and vegetation restoration have gradually become the key tasks of abandoned land reclamation and ecological restoration. In 2014, the abandoned land was leveled and covered mine stripped topsoil, then Korean pine (PKSZ), black locust (RPL), Chinese poplar (PSC) were selected as pioneer species to plant because these species were more adaptable to the local fragile ecological environment and had a high survival rate. Permanent sites had been established in 2014, and an unrestored site was selected for reference. Rhizosphere soil from *Pinus koraiensis* Sieb. et Zucc. (PKSZ), *Robinia pseudoacacia* L (RPL), *Populus simonii* Carr (PSC), and soil from unrestored area (CK) in revegetated iron-mining sites were collected in June 2019. Four plots (20 × 20 m) were randomly established in each site as repetitions, with a distance of approximately 50 m. In each plot, 9 plants with well-grown and consistent growth were randomly selected, then large pieces of sand and other debris on the surface were removed. Fine root samples and soil samples were collected at a depth of 0–30 cm, and the rhizosphere soil of 9 plants at the same plot were collected, mixed as one sample, then placed in a ziplock bag and taken back to the laboratory in ice boxes, resulting in 12 samples. The fresh soil samples were divided into two parts. One part removed stone and plant residues was passed through a 2-mm-autoclaved sieve and immediately put into a 2-ml centrifugal tube and stored at − 80 °C until DNA extraction, and the other part was air-dried and sieved for determination of soil characteristics.

### The determination of soil parameters

The soil pH was assayed in soil: water (w/v) of 1:2.5 H_2_O suspensions following shaking of the samples for 30 min, using a pH meter (Mettler Toledo pH (FE20)). The contents of soil total carbon (TC) and total nitrogen (TN) were determined by an elemental analyzer (Euro Vector EA3000). The concentrations of total phosphorus (TP) and available phosphorus (AP) were measured by spectrophotometer (UV-9000S) after digestion with H_2_SO_4_-HClO_4_ and extracted with 0.5 mol·L^−1^ NaHCO_3_, respectively. The available K (AK) content was determined by atomic absorption spectrometry using1.0 mol·L^−1^ NH_4_OAc as extractant. The concentration of available N (AN) was measured by the alkali solution diffusion method (Lu 1999).

### DNA extraction

The DNA was extracted from 0.5 g of soil using the FastDNA SPIN Kit (MP Biomedicals, Santa Ana, CA, USA), according to the manufacturer’s instructions. Amplification of the nuclear ribosomal DNA from the ITS1 region was performed using the fungal specific primer pair ITS1F and ITS2 (Caban et al. 2018; Nottingham et al. 2018). The first PCR (25 μl total per reaction) contained 2 μl of dNTPs (2.5 mM), 2 μl of DNA template (40–50 ng), 8.75 μl of ddH_2_O, 1 μl (10 uM) of forward and reverse primer, respectively; 5 μl of Q5 reaction buffer (5 ×) and Q5 High-Fidelity GC buffer (5 ×), severally; 0.25 μl (5 U/μl) of Q5 High-Fidelity DNA Polymerase (Deng et al. 2020b). Following the initial denaturation at 95 °C for 5 min; 23 cycles of 95 °C for 30 s, 58 °C for 90 s, 72 °C for 4.5 min; then, final elongation at 72 °C for 10 min. The amplicons were purified and quantified using Agencourt AMPure Beads (Beckman Coulter, Indianapolis, IN) and PicoGreen dsDNA Assay Kit (Invitrogen, Carlsbad, CA, USA). PCR products for sequencing were carried out using an Illumina NovaSeq 6000 sequencing platform at Shanghai Personal Biotechnology Co., Ltd, Shanghai, China. The high-throughput sequencing raw data of fungi were uploaded in the NCBI database with the SRA accession number of PRJNA776422.

### Data analysis

Soil characteristics and soil ECM fungal community diversity among different samples were subjected to ANOVA and means were compared by Tukey’s test (*p* < 0.05). Venn diagram was used to analyze the shared and unique OTUs among different samples in Rstudio with the package of vegan. NMDS was used to compare the difference of ECM fungal beta diversity and carried out using R studio with the packages of vegan, permute, and lattice. LEfSe analysis, namely LDA effect size analysis, can find the species with significant differences in abundance between groups (i.e., Biomaker). Spearman’s correlation coefficients between soil basic characteristics and ECM fungal community diversity and composition were analyzed using SPSS 20.0. The effects of experimental variables on ECM fungal communities in roots were analyzed by canonical correspondence analysis (CCA) using the CANOCO 5.10 software package.

## Results

### Soil characteristics

The concentrations of soil TC (*F* = 26.50, *P* < 0.01), TN (*F* = 13.89, *P* = 0.02), C/N (*F* = 77.52, *P* < 0.01), AN (*F* = 43.33, *P* < 0.01), TP (*F* = 15.98, *P* < 0.01), AP (*F* = 28.53, *P* < 0.01), and AK (*F* = 10.57, *P* < 0.01) significantly differed among PKSZ, RPL, PSC and CK. In addition, significant difference of soil pH was observed (*F* = 22.78, *P* < 0.01) (Table 1). RPL hold the highest contents of TC, TN, TP, and AP with 5.71 g/kg, 0.74 g/kg, 2.89 g/kg, and 20.22 mg/kg, respectively, while, PSC hold the highest AK content with 108.98 mg/kg (Table 1). Soil pH, TC, TN, and TP in CK were 8.17 1.30 g/kg, 0.36 g/kg, and 1.03 g/kg.

**Table 1:** Differences in soil characteristics among PKSZ, RPL, and PSC

	CK	PKSZ	RPL	PSC	F	P
pH	8.17a	7.18b	6.84b	7.00b	22.78	< 0.01
TC (g/kg)	1.30c	3.23b	5.71a	5.48a	26.50	< 0.01
TN (g/kg)	0.36b	0.49b	0.74a	0.64a	13.89	< 0.01
C/N	3.64d	6.64c	7.66b	8.64a	77.52	< 0.01
AN (mg/kg)	15.92c	25.65b	38.92a	46.43a	43.33	< 0.01
TP (g/kg)	1.03c	2.26b	2.89a	2.38b	15.98	< 0.01
AP (mg/kg)	13.70b	14.89b	20.22a	13.30b	28.53	< 0.01
AK (mg/kg)	56.88b	69.88b	94.75ab	108.98a	10.57	< 0.01

*CK*, unrestored area; *PKSZ*, *Pinus koraiensis* Sieb. et Zucc.; *RPL*, *Robinia pseudoacacia* L.; *PSC*, *Populus simonii* Carr. Average value ± standard error (*n* = 12). *TC*, total carbon; *TN*, total nitrogen; *C/N*, C to N ration; *AN*, available nitrogen; *TP*, total phosphorus; *AP*, available phosphorus; *AK*, available potassium. Different lowercase letters in same row indicated the significant difference at 0.05 level

### Sequencing and OTUs

**Fig. 1:**
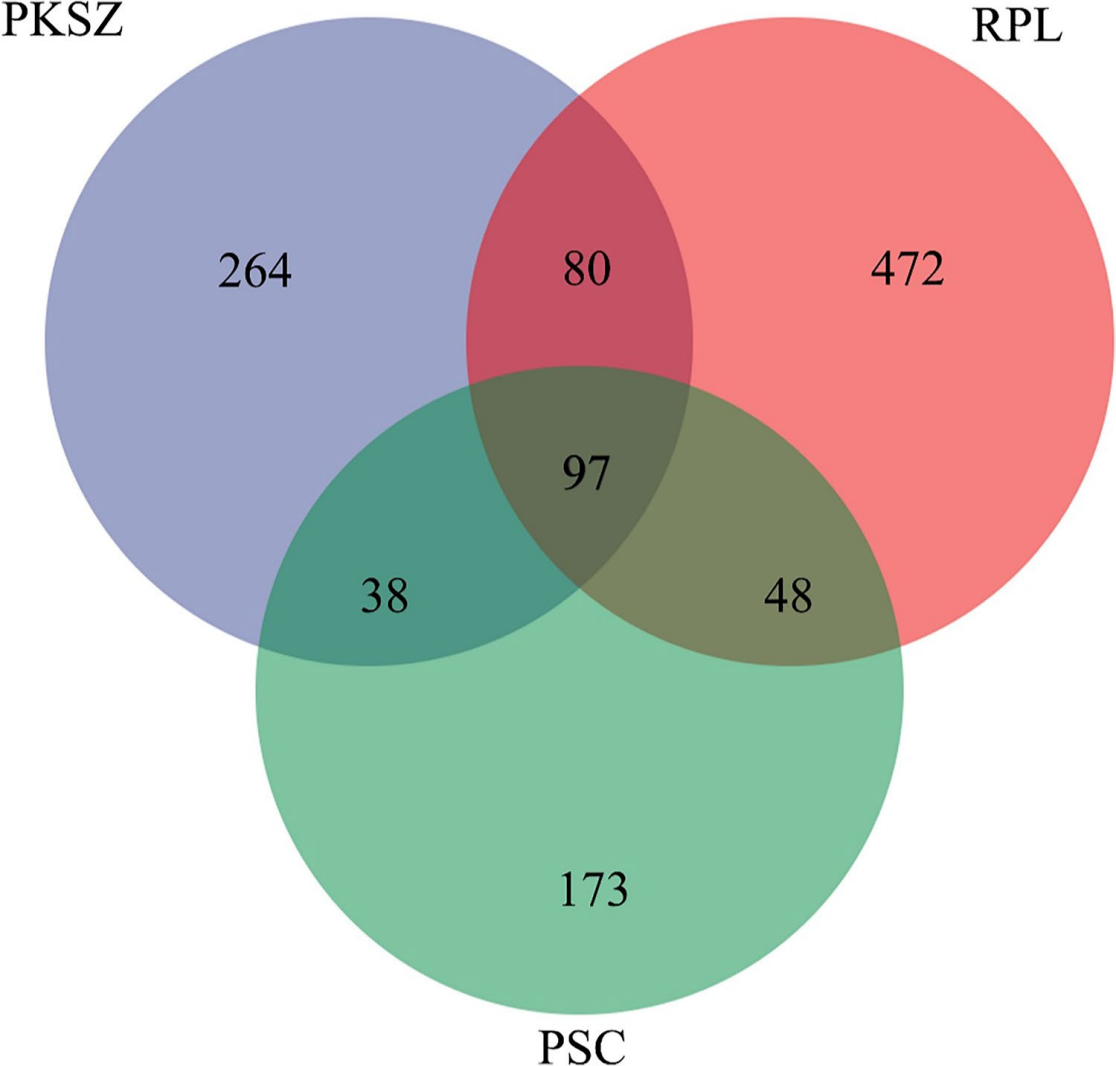
Venn diagram showing shared and unique OTU of PKSZ, RPL, and PSC. PKSZ: *Pinus koraiensis* Sieb. et Zucc., RPL: *Robinia pseudoacacia* L., PSC: *Populus simonii* Carr

A total of 779,651 fungal sequences (reads) were obtained by high-throughput amplification sequencing. After filtering, denoising, merging, removing chimera, and nonsingleton, 659,453 (54,955 per sample) high-quality sequences remained in the dataset, which were clustered into 1172 amplicon sequence variants (ASV) (Fig. 1). As the sequencing depth increased, the rarefaction curves for evaluating the observed_species per sample universally tended to be saturation, demonstrating that the number of sequences was sufficient (Fig. S1). The number of ASVs of PKSZ, RPL, and PSC was 479, 697, and 356, respectively, and the number of ASVs shared by PKSZ, RPL, and PSC was 97 (Fig. 1).

### Ectomycorrhizal fungal diversity

**Fig. 2:**
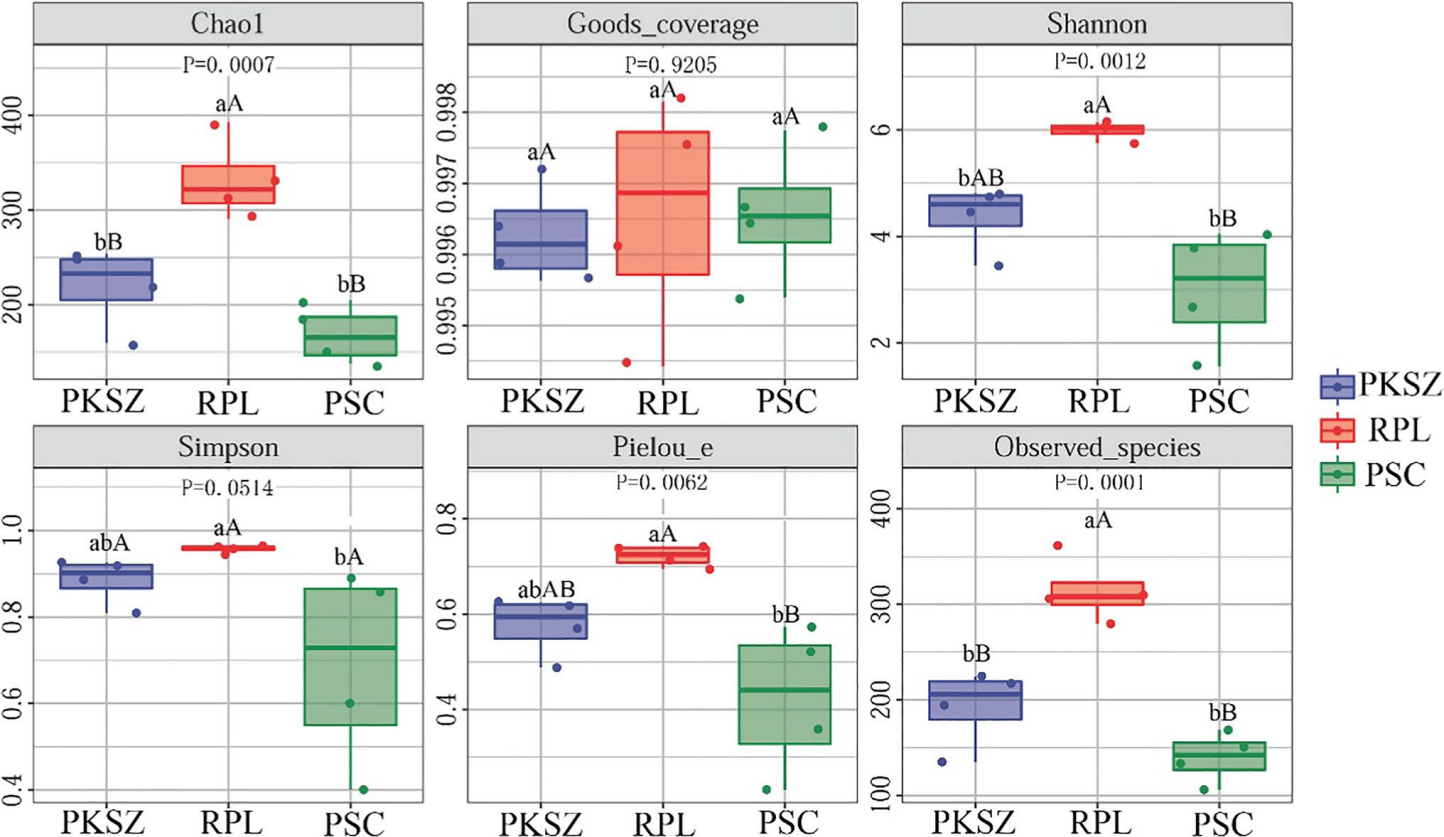
Ectomycorrhizal fungal community diversity among different samples. Different capital letters in same row indicated the significant difference at 0.01 level, and different lowercase letters in same row indicated the significant difference at 0.05. level. PKSZ: *Pinus koraiensis* Sieb. et Zucc., RPL: *Robinia pseudoacacia* L., PSC: *Populus simonii* Carr

Ectomycorrhizal fungal Chao1 index (*F* = 17.98, *P* < 0.01), Pielou_e index (*F* = 9.44, *P* < 0.01), Shannon index (*F* = 15.54, *P* < 0.01), and Observed_species (*F* = 27.37, *P* < 0.01) obviously differed among PKSZ, RPL, and PSC (Fig. 2). In addition, the mean Chao1 index, Pielou_e index, Shannon index, and Observed_species were even greater in RPL than PKSZ and PSC with 331.96, 0.72, 5.99, and 314.35, severally (Fig. 2). Neither Goods_coverage (*F* = 0.08, *P* = 0.92) nor Simpson index (*F* = 4.20, *P* = 0.05) of ECM fungi in roots of PKSZ, RPL, and PSC differed distinctly (Fig. 2). Ectomycorrhizal fungal Chao1 index (*r* = 0.59, *p* < 0.05) and Observed_species (*r* = 0.60, *p* < 0.05) increased with the increase of TP (Table 2). Chao1 index (*r* = 0.76, *p* < 0.01), Observed_species (*r* = 0.78, *p* < 0.01), Pielou_e index (*r* = 0.64, *p* < 0.05), and Shannon index (*r* = 0.70, *p* < 0.05) of ECM fungal communities inhabiting the roots increased with the increase of soil AP (Table 2).

**Table 2:** The relationships between soil characteristics and ectomycorrhizal fungal diversity (*n* = 12)

	PH	TC	TN	C/N	AN	TP	AP	AK
Chao1 index	− 0.31	0.27	0.44	− 0.16	− 0.03	0.59^*^	0.76^**^	− 0.10
Goods_coverage	− 0.03	0.09	0.16	− 0.07	− 0.03	− 0.10	0.03	0.07
Observed_species	− 0.32	0.29	0.48	− 0.17	− 0.04	0.60^*^	0.78^**^	− 0.10
Pielou_e index	− 0.08	0.24	0.46	− 0.30	− 0.25	0.20	0.64^*^	− 0.32
Shannon index	− 0.14	0.25	0.48	− 0.29	− 0.20	0.31	0.70^*^	− 0.27
Simpson index	0.02	0.16	0.35	− 0.30	− 0.35	0.01	0.44	− 0.49

^*^P < 0.05, ** *P* < 0.01. *TC*, total carbon; *TN*, total nitrogen; *C/N*, C to N ration; *AN*, available nitrogen; *TP*, total phosphorus; *AP*, available phosphorus; *AK*, available potassium

### Ectomycorrhizal fungal community composition

**Fig. 3:**
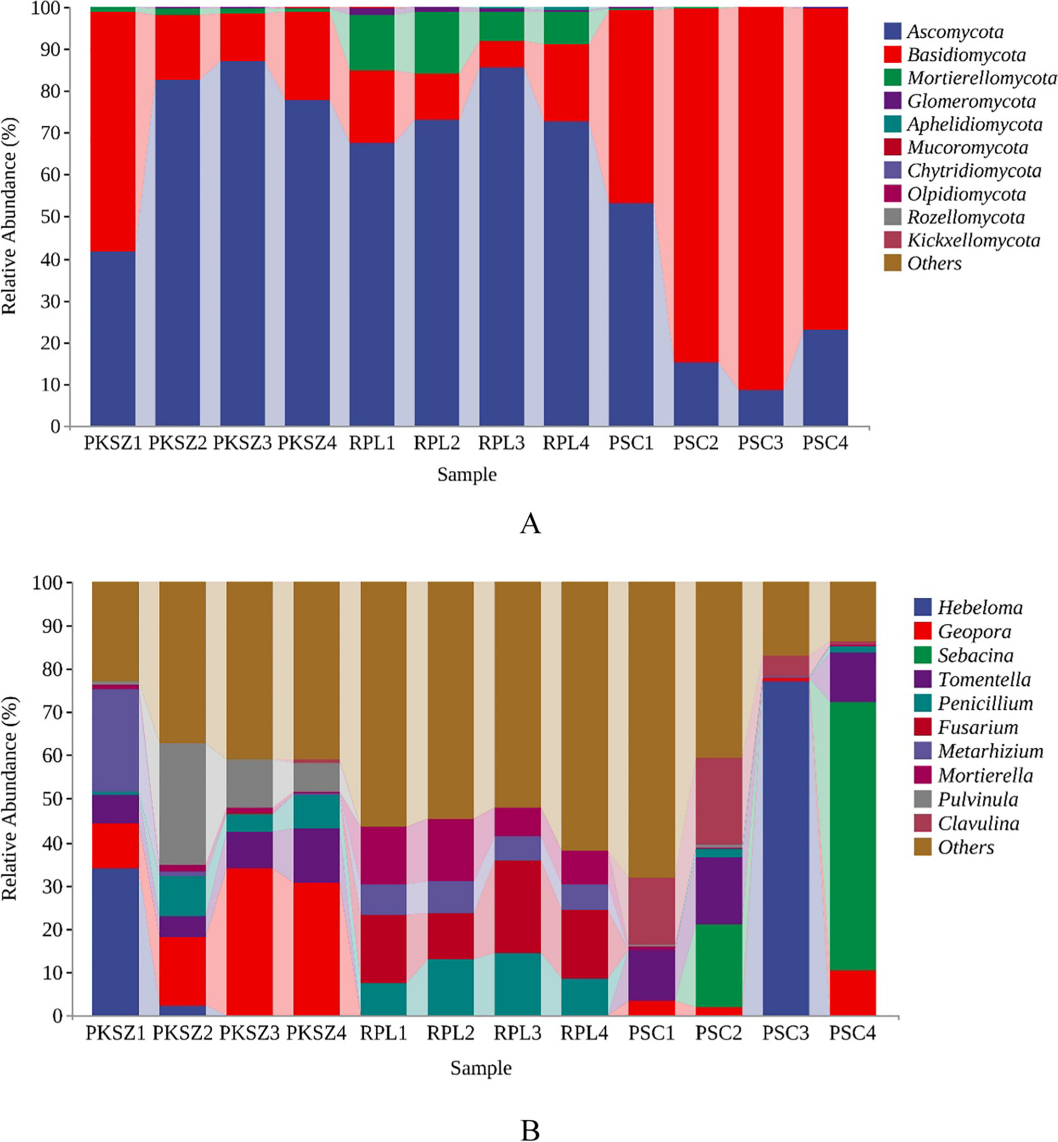
The relative abundance of ectomycorrhizal fungl community at phylum (**A**) and genus (**B**) levels. PKSZ: *Pinus koraiensis* Sieb. et Zucc., RPL: *Robinia pseudoacacia* L., PSC: *Populus simonii* Carr

A total of 12 phyla, 38 classes, 395 genus, and 575 species were identified in our study. The dominant fungal groups were Ascomycota, Basidiomycota, and Mortierellomycota at the phylum level, accounting for 99.43% (Fig. 3A). Agaricomycetes, Pezizomycetes, Sordariomycetes, Eurotiomycetes, Dothideomycetes, Mortierellomycetes, Leotiomycetes, and Tremellomycetes were the dominant fungal groups at the class level (Fig. S2A). At the genus level, the fungal groups with the average relative abundance more than 3.5% were *Hebeloma*, *Geopora*, *Sebacina*, *Tomentella*, *Penicillium*, *Fusarium*, *Metarhizium*, *Mortierella*, *Pulvinula*, and *Clavulina* (Fig. 3B). At the species level, the fungal groups with the average relative abundance more than 2.0% were *Hebeloma_mesophaeum*, *Geopora*_*arenicola*, *Clavulina*_*cinerea*, *Cenococcum*_*geophilum*, *Tomentella*_*ellisii*, *Gibberella*_*baccata*, and *Mortierella*_*alpina* (Fig. S2B).

**Fig. 4:**
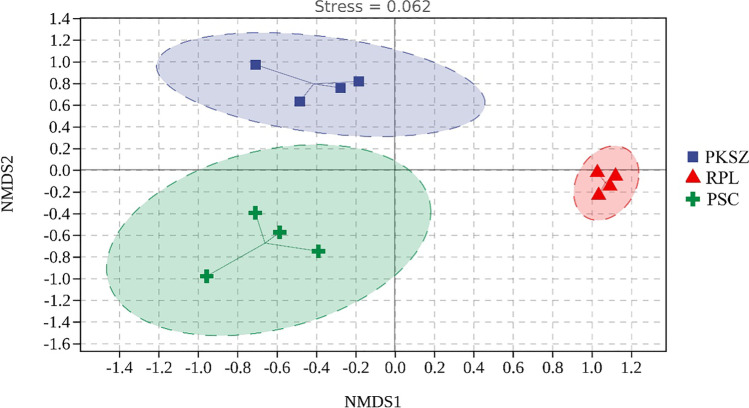
The ectomycorrhizal fungal community beta diversity among different samples. Each point in the figure represents a sample, and points with different colors indicate different samples (groups). Since NMDS adopts rank ordering, it can be approximated that the closer (far) the distance between two points is, the smaller the difference (larger) of the microbial communities in the two samples is. We provide the elliptical dotted circle, which is the 95% confidence ellipse. PKSZ: *Pinus koraiensis* Sieb. et Zucc., RPL: *Robinia pseudoacacia* L., PSC: *Populus simonii* Carr

**Fig. 5:**
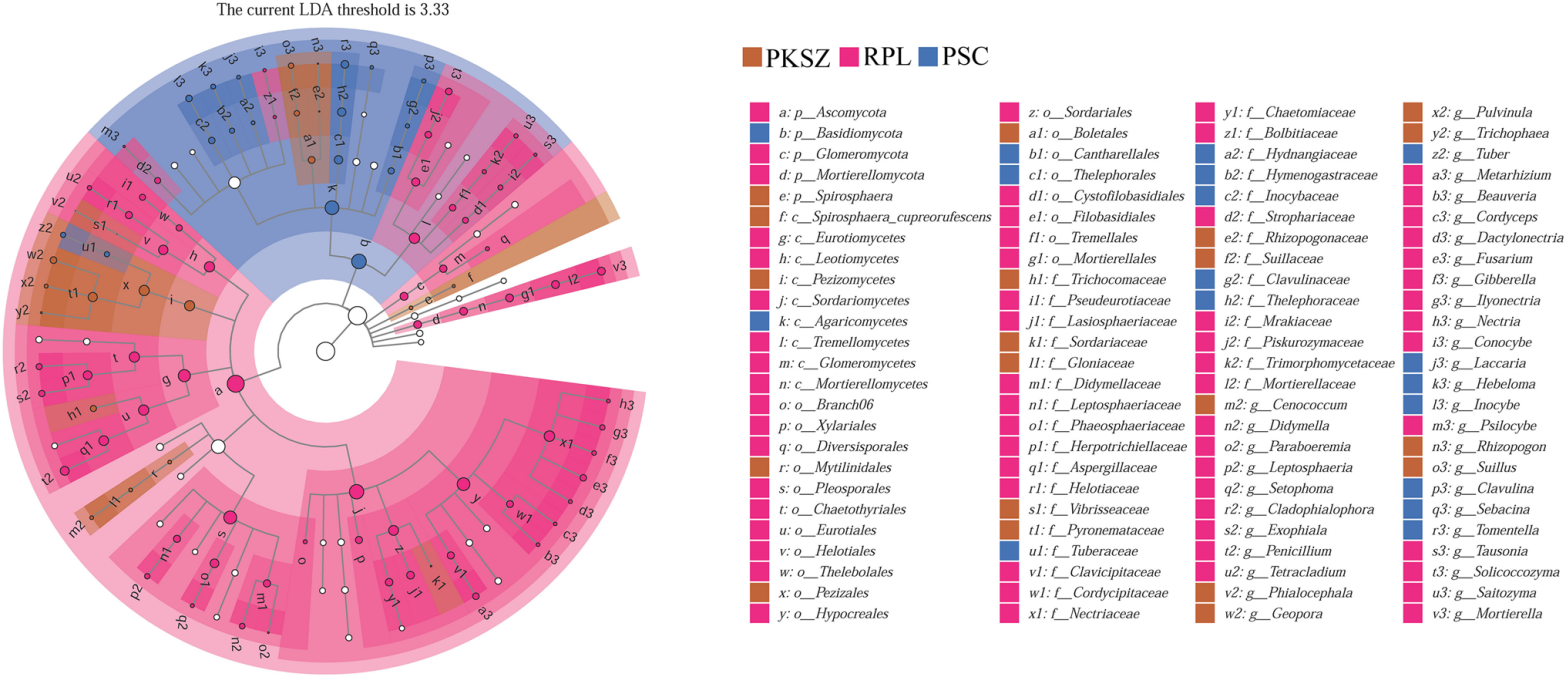
Least discriminant analysis (LDA) effect size taxonomic cladogram. A taxonomic cladogram showing the taxonomic hierarchies of major taxa from phylum to genus (from inner circle to outer circle) in the sample community. Node size corresponds to the average relative abundance of that taxon; hollow nodes represent taxa that are not significantly different between groups, while nodes in other colors (e.g., green and red) indicate that these taxa exhibit significant between-group differences, and abundance is higher in the grouped samples represented by this color. Letters identify the names of taxa that differ significantly between groups. PKSZ: *Pinus koraiensis* Sieb. et Zucc., RPL: *Robinia pseudoacacia* L., PSC: *Populus simonii* Carr

The NMDS (stress = 0.062) demonstrated that ectomycorrhizal fungal community composition clearly differed among PKSZ, RPL, and PSC especially along NMDS1 (Figs. 4). The biomarkers in RPL were Ascomycota (74.72%), Glomeromycota (0.94%), Mortierellomycota (10.64%), Eurotiomycetes (18.04%), Leotiomycetes (2.66%), Sordariomycetes (42.34%), Tremellomycetes (3.52%), *Penicillium* (10.71%), *Fusarium* (16.11%), *Metarhizium* (6.36%), *Mortierella* (10.64%), *Gibberella* (7.01%), and *Didymella* (3.34%) (Fig. 5). The biomarkers in PKSZ were Pezizomycetes (15.68%), *Geopora* (22.55%), *Suillus* (5.52%), *Pulvinula* (12.71%), and *Cenococcum* (9.25%) (Fig. 5). The biomarkers in PSC were Basidiomycota (74.67%), Agaricomycetes (25.925), *Laccaria* (4.41%), *Hebeloma* (19.29%), *Inocybe* (3.47%), *Sebacina* (20.31%), *Tomentella* (9.70%), and *Clavulina* (10.26%), and *Tuber* (9.45%) (Fig. 5).

### Contribution of soil properties to ectomycorrhizal fungal community composition

**Fig. 6:**
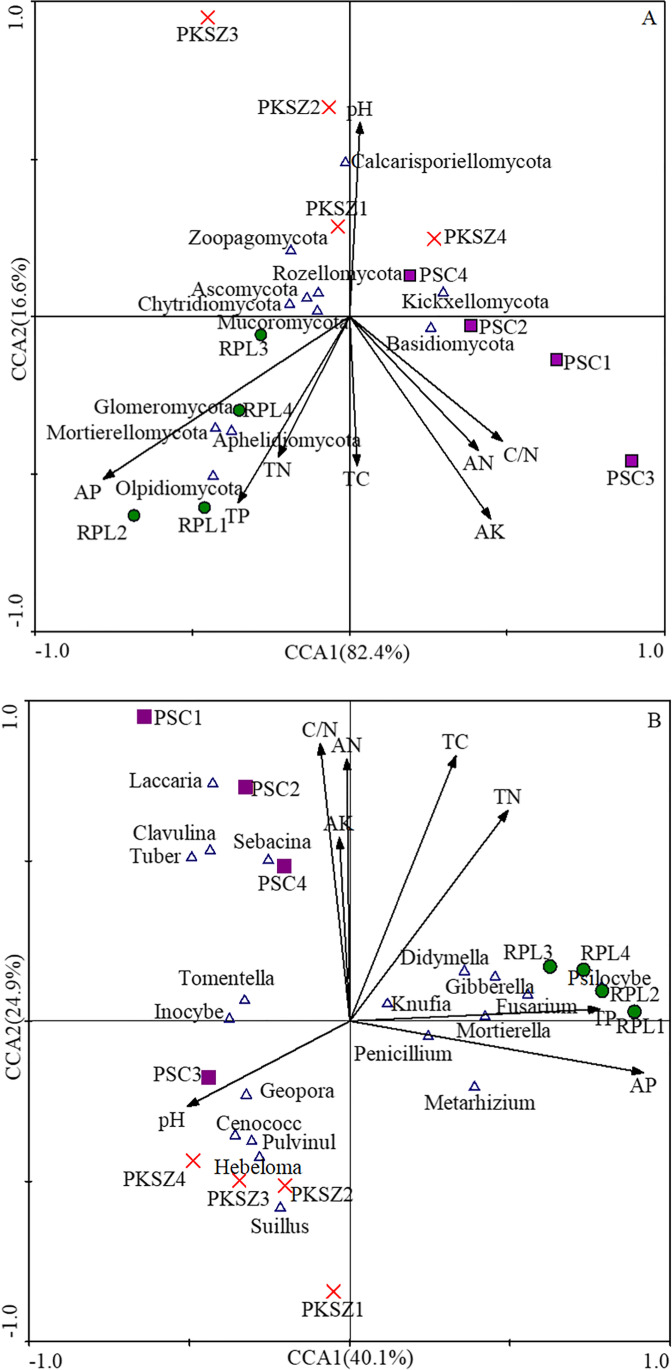
The contribution of soil properties to ectomycorrhizal fungal community composition at the phylum (**A**) and genus (**B**) level. Different shapes represent different samples; blue triangles in the figure represent different bacteria. The angle between species and environmental factors represents the positive and negative correlations between species and environmental factors. Vertical lines are drawn from different samples to each environmental factor, and the closer the projection points are, the more similar the attribute values of the environmental factor between the samples are. That is, the environmental factors have the same degree of influence on the samples. PKSZ: *Pinus koraiensis* Sieb. et Zucc., RPL: *Robinia pseudoacacia* L., PSC: *Populus simonii* Carr. TC, total carbon; TN, total nitrogen; C/N, C to N ration; AN, available nitrogen; TP, total phosphorus; AP, available phosphorus; AK, available potassium

For the ectomycorrhizal fungal community at the phylum level, all the eight soil characteristics explained 99.9% of the variance, with axis 1 explaining 82.40% of the variance and axis 2 explaining 16.60% (Fig. 6A). For the fungal community at the genus level, all the eight soil characteristics explained 65.0% of the variance (Fig. 6B), with axis 1 explaining 40.10% of the variance and axis 2 explaining 24.90% (Fig. 6B). Notably, the concentration of AP in soil was positively correlated with Mortierellomycota (*r* = 0.68, *p* < 0.05) and Glomeromycota (*r* = 0.59, *p* < 0.05). Aphelidiomycota was significantly positive correlation with the concentration of soil TN (*r* = 0.59, *p* < 0.05), TP (*r* = 0.77, *p* < 0.01), and AP (*r* = 0.90, *p* < 0.01), while Mortierellomycota (*r* = − 0.73, *p* < 0.01), Glomeromycota (*r* = 0.65, *p* < 0.05), and Aphelidiomycota (*r* = − 0.75, *p* < 0.01) decreased with the increase of soil pH (Table 3).

**Table 3:** The relationship between soil basic characteristics and ectomycorrhizal fungal community composition. (*n* = 12)

	pH	TC	TN	C/N	AN	TP	AP	AK
Ascomycota	-0.17	-0.12	0.01	-0.52	-0.40	-0.11	0.22	-0.58^*^
Basidiomycota	0.47	− 0.07	− 0.24	0.34	0.29	− 0.15	− 0.47	0.52
Mortierellomycota	− 0.73^**^	0.11	0.29	− 0.28	− 0.35	0.48	0.69^*^	− 0.53
Glomeromycota	− 0.65^*^	0.29	0.39	− 0.06	− 0.13	0.30	0.59^*^	− 0.34
Aphelidiomycota	− 0.75^**^	0.49	0.59^*^	0.15	0.27	0.77^**^	0.90^**^	0.03
	pH	TC	TN	C/N	AN	TP	AP	AK
*Hebeloma*	0.55	− 0.78^**^	− 0.81^**^	− 0.46	− 0.33	− 0.28	− 0.37	− 0.05
*Geopora*	0.75^**^	− 0.65^*^	− 0.69^*^	− 0.43	− 0.41	− 0.69^*^	− 0.64^*^	− 0.21
*Sebacina*	0.51	− 0.27	− 0.40	0.16	0.04	− 0.41	− 0.76^**^	0.14
*Tomentella*	0.79^**^	− 0.17	− 0.29	0.05	0.00	− 0.74^**^	− 0.74^**^	0.25
*Penicillium*	− 0.57	0.18	0.38	− 0.36	− 0.14	0.13	0.43	− 0.28
*Fusarium*	− 0.68^*^	0.29	0.43	− 0.22	− 0.17	0.42	0.61^*^	− 0.51
*Metarhizium*	− 0.61^*^	− 0.24	− 0.06	− 0.48	− 0.58^*^	0.55	0.77^**^	− 0.62^*^
*Mortierella*	− 0.73^**^	0.11	0.29	− 0.28	− 0.35	0.48	0.70^*^	− 0.53
*Pulvinula*	0.57	− 0.61^*^	− 0.63^*^	− 0.64^*^	− 0.68^*^	− 0.67^*^	− 0.51	− 0.47
*Clavulina*	0.63^*^	0.28	0.07	0.44	0.46	− 0.53	− 0.60^*^	0.70^*^

^*^P < 0.05, **P < 0.01. *TC*, total carbon; *TN*, total nitrogen; *C/N*, C to N ration; *AN*, available nitrogen; *TP*, total phosphorus; *AP*, available phosphorus; *AK*, available potassium

At the genus level, soil fungal community composition was driven by soil properties (Table 3). The relation abundance of *Hebeloma* (*r* = − 0.78, p < 0.01; *r* = − 0.81, *p* < 0.01) and *Geopora* (*r* = − 0.65, *p* < 0.05; *r* = − 0.69, *p* < 0.05) declined with the increase of TC and TN. There were high correlation coefficients present between AP and Geopora (*r* = − 0.64, *p* < 0.05), Sebacina (*r* = − 0.76, *p* < 0.01), Tomentella (*r* = − 0.74, *p* < 0.01), Fusarium (*r* = 0.61, *p* < 0.05), Metarhizium (*r* = 0.77, *p* < 0.01), Mortierella (*r* = 0.70, *p* < 0.05), Clavulina (*r* = -0.60, *p* < 0.05) (Table 3).

## Discussion

### Responses of soil characteristics to different revegetation

In present study, it was found that the soil nutrient contents increased significantly after 6 years of vegetation restoration in Anshan-Benxi iron-mined area, in Liaoning Province, indicating that the implementation of ecological engineering was beneficial soil carbon sequestration, which is consistent with other studies (Zhang et al. 2019; Hu et al. 2018). On the one hand, no mining activities can promote the formation of soil aggregates, thereby improving the SOC holding capacity. On the other hand, the increase in litter and root exudates after vegetation restoration increases the source of carbon input (Hong et al. 2021). It follows that the decrease of organic carbon mineralization and the increase of carbon input sources were the main reasons for the increase of SOC after vegetation restoration. Also, significant differences in the concentrations of soil TC, TN, C/N, AN, TP, AP, and AK differed significantly among PKSZ, RPL, and PSC (Table 1), which were highly similar to those reported by Xu et al. (2014). What’s more, compared to PKSZ and PSC, RPL could better improve soil TC, TN, TP, and AP (Table 1), which was consistent with previous studies demonstrated that broadleaf forest could improve soil nutrients than coniferous forest (Deng et al. 2019a, b). As we all know, the turnover of litter and fine roots is the main way of soil organic matter input, and the content of organic matter can affect the process of nitrogen transformation and accumulation. The relatively high soil organic matter and total nitrogen content of RPL may be related to factors such as higher litter content such as litter and stronger root replacement. Moreover, rhizobium related to the roots of *Robinia pseudoacacia* can fix nitrogen in the atmosphere and enrich soil nitrogen (Li et al. 2018). Apart from low nutrients, the soil pH in unrestored area here was alkaline, and neared neutrality in the presence of plants, which could improve the bioavailability of essential micronutrients.

### Responses of soil ectomycorrhizal fungal community diversity and composition to different revegetation

An increasing body of research has shown that soil microorganisms are more sensitive than soil characteristics and can rapidly respond to environmental changes (Munoz-Rojas et al. 2016). Soil microbial biomass, community diversity and composition as well as function are potential biological indicators of soil quality (Dinesh and Chaudhuri 2013), which are applied to monitor the restoration of soil ecosystem functions during the restoration process in different degraded ecosystems (Sun et al. 2016; Yu et al. 2016). In present study, we compared the difference of soil ectomycorrhizal fungal community diversity and composition among three different vegetation restoration types, and linked the changes in the microbial combination with the soil properties.

Vegetation restoration and reconstruction regulate the interaction between microbial community and forest development, which is mainly manifested in the dynamic changes of microbial diversity and structure (Chanthorn et al. 2017). Our findings generally suggested that soil ectomycorrhizal fungal community diversity varied with vegetation restoration, and RPL hold the highest ectomycorrhizal fungal Chao1 index, Pielou_e index, Shannon index, and Observed_species (Fig. 2). This finding is coherent with the results of Deng et al. (2020a, b). Soil microorganisms participate in a series of soil biochemical processes, which are closely related to the conversion of soil organic carbon (Rallage et al. 2021). In the process of vegetation restoration, a large amount of exogenous carbon entering the soil will be decomposed by soil carbon degrading enzymes to release low-molecular-weight sugars, providing important carbon and energy sources for microbial growth and metabolism (Davidson et al. 2004), thereby increasing soil microbial community diversity.

Soil ectomycorrhizal fungal diversity reveals that the revegetation process plays an important role in the development of the microbial community composition. The results showed that overall ectomycorrhizal fungal community structure differed significantly among three different vegetation (Fig. 5), which supported our second hypothesis, confirming previous results which showed that ECM community structures may be directly impacted by their host (Sugiyama et al. 2021; Rosinger et al. 2018; Scheibe et al. 2015; Urbanová et al. 2015; Saitta et al. 2018; Molina and Horton 2015). Differences in soil microbiome among different samples were mainly caused by the plant community, as observed in other mining site under a revegetation program. These results confirmed that different components of the root microbiome can be complementary in the acquisition of essential and limiting nutrients in the ecosystem (Patricia et al. 2016).

In our study, the predominant ectomycorrhizal fungal group was Ascomycota, which was consistent with previous study (Guo et al. 2018). Ascomycota were detected in all sites, which degrade cellulose and more complex carbohydrates in the litter (Schoch et al. 2006) and adapt to nutrient-poor and dry habitats (Ruibal et al. 2009). The predominance of the Ascomycota phylum, followed by Basidiomycota, is common in forests (Jesús et al. 2017). This information suggests that PKSZ, RPL, and PSC areas are recovering their ecosystem functions. The predominance of certain fungi genera in the soil interacting with certain plant species can ensure functional redundancy in different ecological contexts (Louca et al. 2018). *Russula*, *Cortinarius*, *Tomentella*, and *Tuber* were the predominant ectomycorrhizal fungal groups of *Quercus liaotungensis* from Dongling Mountain, Beijing (Wang et al. 2012). In addition, *Russula* was the core ectomycorrhizal fungal group of *Quercus variabilis* in Taihang Mountain gneiss area (Wei et al. 2018). The main ectomycorrhizal fungi of *Quercus falciparum* in Shangyu beach, Zhejiang province were *Russula* and *Tomentella* (Wei et al. 2020). It can be seen that the main ectomycorrhizal fungal groups of different tree species and regions are different, which is closely related to the characteristics of tree species and environmental factors. Collectively, these studies indicated the diversity and composition of ectomycorrhizal fungi could be used as an important index for evaluating the restoration of soil functions.

### The relationships between soil characteristics and ectomycorrhizal fungal community

Besides differences in host, abiotic conditions formed another important filter for ectomycorrhizal fungal communities. Soil fungal community plays an important role in biogeo-chemical cycles in ecosystems and can be significantly affected by environmental disturbances (Jesús et al. 2017). The integration of soil biological indicators with chemical and physical indicators is an important factor in the evaluation of soil quality and the recovery process (Silva et al. 2018). Regarding the third results, the effects of environmental factor and host on pattern of ECM fungal community structure at the regional scale have been speculated in previous studies (Tedersoo et al. 2012; Wu et al. 2018b). Acidification and increasing the availability of nitrogen have a strong impact on ectomycorrhizal fungal community diversity, richness, and evenness (Toljander et al. 2006; Suz et al. 2015), while no similar findings were obtained in our study. As a result, soil TP and AP were the main factors effecting soil ectomycorrhizal fungal community diversity, especially Chao1 index, Observed_species, Pielou_e index, and Shannon index.

Soil microbes should be considered drivers of productivity diversity in terrestrial ecosystems. In the process of vegetation restoration, soil fungal community was significantly affected by the changing soil properties (Zak and Cline 2015), which in turn, were most likely affected by vegetation (Yao et al. 2018; Barnes et al. 2018). What’s more, previous research also has already shown that soil pH is considered a most important factor in shaping soil ectomycorrhizal fungal community composition (Kutszegi et al. 2015; Matsuoka et al. 2016), and it was confirmed in our study. Soil pH cannot only directly affect the community composition of ectomycorrhizal fungi, because the optimum pH value of ectomycorrhizal fungi is different, but also indirectly affect the community composition of ectomycorrhizal fungi by affecting nutrient availability (Erland and Taylor 2002), while some findings demonstrated that no significant influence of soil pH on soil microbial community composition (Yu et al. 2020; Bastida et al. 2013). This may be due to the low sensitivity of fungi and the wide optimum range of soil conditions (Rousk et al. 2010), or the significant fluctuation of soil pH with vegetation restorations. Soil fungi perform important functions in nutrient cycling, while soil nutrients shape soil fungal communities with different functional groups (Li et al. 2018). In our study, soil TC, TN, TP, AP had important roles in the soil fungal community, which was consistent with a previous large-scale research study (Schappe et al. 2020; Cai et al. 2018). It follows then that the differences in ectomycorrhizal fungal diversity and composition found between samplings could be attributed to the different revegetation types.

## Conclusion

In conclusions, the current study has uncovered the distinct difference of soil characteristics and ectomycorrhizal fungal community composition in a typical Fe ore tailing in Liaoning. It is noteworthy that soil properties could be improved by different revegetation types, and RPL could significantly better improve soil nutrients than PKSZ and PSC. In addition, compared to PKSZ and PSC, RPL could better improve soil ectomycorrhizal fungal community diversity. Soil ectomycorrhizal fungal community composition significantly differed depending on revegetation types. Changes of soil nutrients caused by different revegetation types were key factors affecting the ectomycorrhizal fungal community diversity and composition. Thus, these results indicated that RPL might be a more suitable species for the revegetation of iron mine tailings.

## Supplementary Information

Below is the link to the electronic supplementary material.

Supplementary file1 (DOCX 471 KB)

## Data Availability

The datasets used and analyzed during the current study are available from the corresponding author on reasonable request.
