# Supplementary material for: Characterization of ectomycorrhizal fungal communities associated with tree species on an iron tailings deposit undergoing restoration
Source: Environ Sci Pollut Res Int. 2022 Jul 2;29(56):84396–409. doi: 10.1007/s11356-022-21690-0 (PMC9646614; doi:10.1007/s11356-022-21690-0)
Supplement: Supplementary file 1 — Supplementary file1 (DOCX 471 KB) [file 11356_2022_21690_MOESM1_ESM.docx]

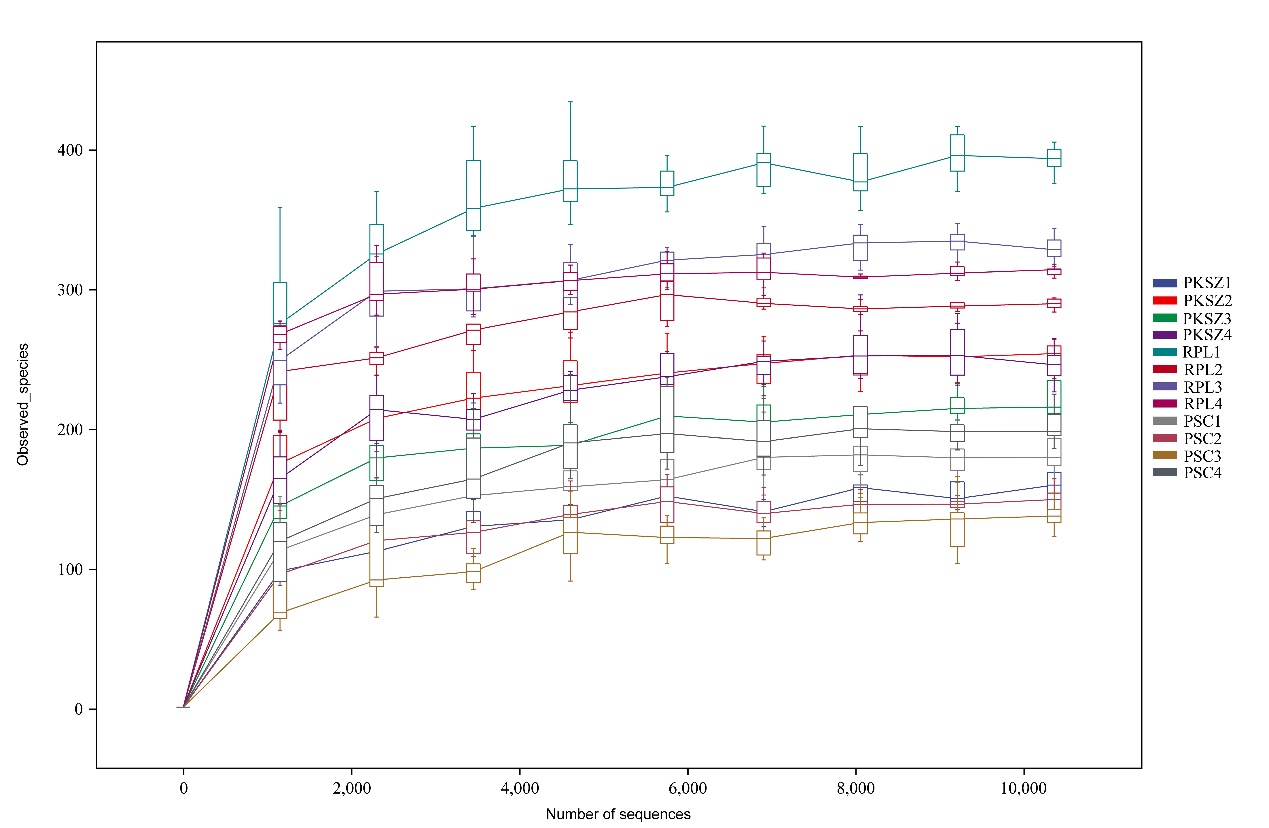


Figure S1 rarefaction curves. PKSZ: *Pinus koraiensis* Sieb. et Zucc., RPL: *Robinia pseudoacacia* L., PSC: *Populus simonii* Carr.


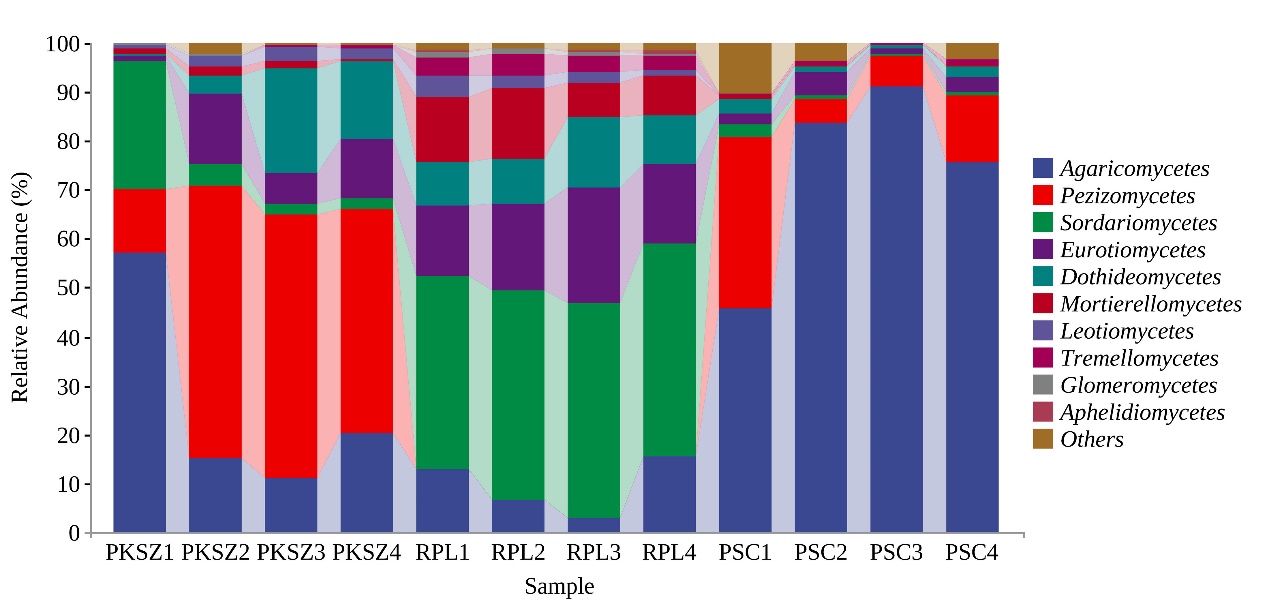


A


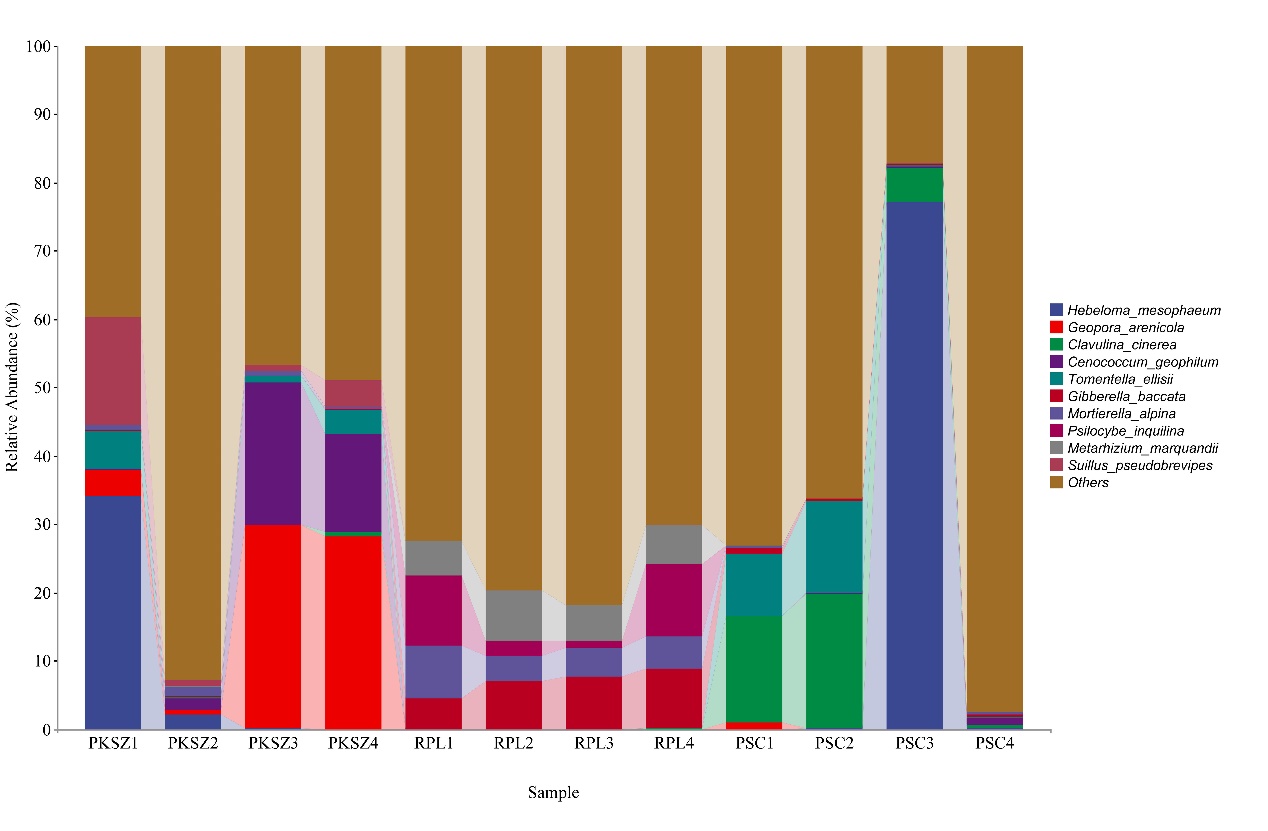


B

Figure S2The relative abundance of ectomycorrhizal fungi at the class (A) and species (B) levels. PKSZ: *Pinus koraiensis* Sieb. et Zucc., RPL: *Robinia pseudoacacia* L., PSC: *Populus simonii* Carr.
